# Functional evaluation of therapeutic response of HCC827 lung cancer to bevacizumab and erlotinib targeted therapy using dynamic contrast-enhanced and diffusion-weighted MRI

**DOI:** 10.1371/journal.pone.0187824

**Published:** 2017-11-09

**Authors:** Yi-Fang Chen, Ang Yuan, Kuan-Hung Cho, Yi-Chien Lu, Mark Yen-Ping Kuo, Jyh-Horng Chen, Yeun-Chung Chang

**Affiliations:** 1 Graduate Institute of Clinical Dentistry, National Taiwan University, Taipei, Taiwan; 2 Department of Internal Medicine, National Taiwan University College of Medicine, Taipei, Taiwan; 3 Institute of Biomedical Engineering and Nanomedicine, National Health Research Institutes, Miaoli, Taiwan; 4 Department of Medical Imaging, National Taiwan University Hospital and National Taiwan University College of Medicine, Taipei, Taiwan; 5 Interdisciplinary MRI/MRS Lab, Department of Electrical Engineering, National Taiwan University, Taipei, Taiwan; Catalan Institute of Oncology, SPAIN

## Abstract

This study aimed to investigate the therapeutic responses of lung cancer mice models with adenocarcinoma HCC827 (gefitinib sensitive) and HCC827R (gefitinib resistant) to the epidermal growth factor receptor-tyrosine kinase inhibitor erlotinib alone and in combination with the anti-angiogenesis agent bevacizumab using dynamic contrast enhanced (DCE) and diffusion-weighted MRI. In the HCC827 model, temporal changes in DCE-MRI derived parameters (*K*^*trans*^, *k*_*ep*_, and *iAUC*_*90*_) and apparent diffusion coefficient (ADC) were significantly correlated with tumor size. *K*^*trans*^ and *iAUC*_*90*_ significantly decreased at week 2 in the groups receiving erlotinib alone and in combination with bevacizumab, whereas *k*_*ep*_ decreased at week 1 and 2 in both treatment groups. In addition, there was a significant difference in *iAUC*_*90*_ between the treatment groups at week 1. Compared to the control group of HCC827, there was a significant reduction in microvessel density and increased tumor apoptosis in the two treatment group. ADC value increased in the erlotinib alone group at week 1 and week 2, and in the erlotinib combined with bevacizumab group at week 2. Enlarged areas of central tumor necrosis were associated with a higher ADC value. However, progressive enlargement of the tumors but no significant differences in DCE parameters or ADC were noted in the HCC827R model. These results showed that both erlotinib alone and in combination with bevacizumab could effectively inhibit tumor growth in the gefitinib-sensitive lung cancer mice model, and that this was associated with decreased vascular perfusion, increased ADC percentage, decreased microvessel density, and increased tumor apoptosis with a two-week treatment cycle.

## Introduction

Lung cancer is the most common cause of cancer-related death worldwide, and it is a significant public health problem associated with a poor prognosis under current therapeutic options [1]. Recent advances in cancer therapeutics have shown that identifying molecular targets for lung cancer treatment such as vascular endothelial growth factor (VEGF) and epidermal growth factor receptor (EGFR) is important to improve patient survival. VEGF is a pro-angiogenic factor which binds membrane receptors, and its intra-cytoplasm domain presents tyrosine kinase activity. Angiogenesis is considered to be important for tumor growth, metastasis and patient prognosis [2–4]. Targeted therapy against angiogenesis can lead to regression or normalization of neovascular structures, and the inhibition of new blood vessel growth. Bevacizumab (Avastin, Genentech/Roche) is a monoclonal antibody that selectively binds to VEGF and prevents interactions with its receptor. It was shown to significantly prolong progression-free survival (PFS) and overall survival when added to doublet chemotherapy in Eastern Cooperative Oncology Group (ECOG) study E4599, and to prolong PFS in the Avastin in Lung (AVAiL) study. Bevacizumab is approved for patients with advanced non-squamous non-small cell lung cancer (NSCLC) [5–7]. However, responses to bevacizumab alone have been reported to be transient and susceptible to resistance, so that other targets for the treatment of patients with NSCLC are needed [8].

Somatic mutations in the EGFR gene have been identified in patients who respond to EGFR-tyrosine kinase inhibitors (TKIs) [9–11]. The two most common EGFR mutations are deletions of exon 19 that eliminate a leucine-arginine-glutamate-alanine motif in the tyrosine kinase domain of EGFR, and L858R point mutations in exon 21 that result in substitution of arginine for leucine at amino acid 858 [12]. Gefitinib and erlotinib target the EGFR tyrosine kinase detectable in most cases of NSCLC. Phase II and III trials have shown partial responses in 8% to 12% of patients with progressive NSCLC after chemotherapy [13–16]. In addition, a phase II study suggested synergistic activity in first-line treatment with a combination of bevacizumab and erlotinib in stage IIIb/IV NSCLC [17], and the addition of bevacizumab to erlotinib has been reported to prolong PFS in patients with NSCLC with activating EGFR mutations compared with erlotinib alone [18]. The phase II trial (BELIEF) also provided evidence of benefit for the combination of erlotinib and bevacizumab in patients with EGFR-mutant NSCLC [19].

Research into the mechanism and therapeutic response of lung cancer to variable treatment strategies and the development of new treatment paradigms are of the utmost importance. The current clinical standard to evaluate tumor response is based on the response evaluation criteria in solid tumors (RECIST), which define the treatment response only in terms of tumor size [20]. However, using size alone to assess tumor response has limitations, and in particular when assessing newer cancer therapies aimed at stabilizing the disease [21–23]. It is known that a change in the size of a tumor may be delayed chronologically, and thus several courses of treatment are often required before a conclusion can be made regarding a treatment response [23]. Recent studies have demonstrated that a multi-parametric imaging approach combining information from different functional imaging techniques such as dynamic contrast-enhanced (DCE) magnetic resonance imaging (MRI), diffusion-weighted (DW)-MRI, susceptibility MRI, and perfusion computed tomography (CT), is a promising approach to assess treatment response in the early phases of drug development [24–26]. DCE-MRI provides important pharmacokinetic parameters which can measure sensitive pathophysiological characteristics and detect changes in tumor vasculature and the peritumoral microenvironment [27–28]. For example, increased tumor angiogenesis may increase tumor perfusion and blood volume, which can be detected and quantified by using DCE-MRI [29]. In addition, tumor necrosis and apoptosis may decrease cellularity and change the peritumoral microenvironment which can be quantified by using DW-MRI [30]. DW-MRI generates an apparent diffusion coefficient (ADC) map which has been shown to correlate with cell density measures in cancer models [31– 32].

A previous study reported a decrease in tumor size and decreased values of all MR-derived pharmacokinetic parameters in patients receiving the anti-angiogenic agent bevacizumab in combination with gemcitabine and cisplatin as first-line treatment, and therefore MR parametric histograms may have the potential to predict an early treatment response to such treatment [33]. Recently, target therapy alone or in combination with chemotherapy has been used to treat advanced NSCLC with/without distant metastasis [7, 15, 34–37]. Consequently, treatment with bevacizumab and erlotinib targeting different tumor growth pathways has become a new therapeutic option for lung cancer. On the basis of little overlap in their toxic-effect profiles, the aim of this study was to assess the therapeutic response of lung cancer to bevacizumab and erlotinib treatment using DCE and DW-MRI in a mice model.

## Materials and methods

### Lung cancer cell lines, drugs and animal preparation

In NSCLC, mutations in the EGFR tyrosine kinase domain have been associated with sensitivity to erlotinib and gefitinib [38]. Therefore, in this study we used the gefitinib hypersensitive NSCLC cell line HCC827 with an exon 19 EGFR deletion, and the acquired resistant cell line HCC827R (gefitinib resistant) [39]. Erlotinib (oral, 25 mg/kg/day) and bevacizumab (i.p., 5 mg/kg/twice a week) were used to treat lung cancer xenografts on the subcutaneous tissue of mice.

### *In vivo* tumorigenesis assay in the murine xenograft model

Severe combined immunodeficiency (SCID) mice were purchased at ages 4 to 6 weeks from the BioLASCO (Taipei, Taiwan) and housed in an isolator and fed with autoclaved food *ad libitum*. For tumor growth, cancer cells were trypsinized, washed, centrifuged, and resuspended. A total volume of 0.1 mL containing 4×10^6^ cells was subcutaneously injected into the dorsal region of each mouse. All of the mice were evaluated using DCE and DW-MRI at the 2^nd^ (baseline), 3^rd^ and 4^th^ weeks after tumor implantation. After the final MR study, mice were anesthetized with intraperitoneal injection of 1.2% Avertin and then sacrificed by intravenous injection of potassium chloride (2 meq/kg). The tumors were confirmed by histological examinations, and the excised tissues were fixed and embedded in ornithine carbamyl transferase. From each frozen block, three consecutive sections were obtained for hematoxylin and eosin (H&E), microvessel and apoptosis staining. All animal experiments were performed in triplicate and all procedures for animal experimental protocols were approved by the Institutional Animal Care and Use Committee of National Taiwan University College of Medicine.

### MR imaging

All animal studies were performed using a 7T animal scanner (BioSpec 70/30 USR, Bruker, Germany) equipped with a surface coil (Bruker). DCE-MR was performed using the following parameters: repetition time (TR) 100.1 ms, echo time (TE) 3.8 ms, flip angle 40 degrees, number of excitations (NEX) 1, 9 slices, field of view 3.5 x 3.5 cm, slice thickness 1 mm, matrix size 256 x 192, resolution 137×182 μm, scan time 14.4 s/acquisition. There were 60 acquisitions and an intraorbital contrast injection was given from the beginning of the 5th scan (i.e. 72 s after the first acquisition).

One b0 image and three DW images in read, phase, and slice gradient directions were acquired using a pulsed-gradient spin-echo segmented echo-planar imaging sequence with the following parameters: TR 3000 ms, TE 29.3 ms, in-plane resolution 273×273 μm, slice thickness 1–1.5 mm, no intersection gap, number of segments 4, NEX 5, and b value 700 s/mm^2^. ADC maps were generated from the DW images. For DW-MRI, 9 slices were acquired at the same location as DCE-MRI.

The mean diffusivity (MD) was then calculated as:

$$MD=\frac{1}{3}\sum_{i}D_{i},$$

where i = read, phase, or slice. D_i_ is the ADC value measured in each direction and calculated as:

$$D_{i}=\frac{\text{ln}\;S_{0}-\text{ln}\;S_{i}}{b},$$

where S_0_ is the MR signal of the b0 image, S_i_ is the MR signal of DWI measured in a given direction, and b is the diffusion sensitivity. To measure the MD inside the tumor, the tumor was first manually selected directly on the MD map by a researcher familiar with the anatomy of mice. After the region of the tumor had been selected, the core region of the tumor was generated by applying morphological erosion to manually identify the region of the tumor iteratively. The iteration stopped when the ratio area of eroded region of interest to the area of non-eroded region of interest was equal to or less than 1/4. The peripheral region of the tumor was defined as the region of the tumor excluding the core region. Mean values of MD in the whole tumor region, and core and peripheral regions of the tumor were then evaluated.

### MR imaging analysis

Analysis of DCE-MRI was carried out using a two-compartment Tofts model [33] and commercial software (Apollo Medical Imaging Technology Pty Ltd, Melbourne, Australia). The parameters derived from the dynamic data included the volume transfer constant *K*^trans^ (min^-1^/1000), rate constant *k*_ep_ (min^-1^), extracellular extravascular space (EES) volume fraction (*v*_e_) (0 < *v*_e_ < 1, dimensionless), and plasma volume fraction (*v*_p_) (0 < *v*_p_ < 1, dimensionless) [27]. In addition, model free curve analysis and parametric histogram analysis [28, 33] were performed. The parameters of conventional curve analysis included maximum enhancement, time to peak (min), uptake rate (min^-1^), and washout rate (min^-1^) as well as area under the curve within 90 seconds (*iAUC*_*90*)_. The quantitative summary statistics of parametric histograms, including the mean, standard deviation (SD), kurtosis and skewness of these parameters (i.e., *K*^trans^ mean, *K*^trans^ SD, *k*_ep_ mean, *k*_ep_ SD, *v*_e_ mean, *v*_e_ SD, *v*_p_ mean, and *v*_p_ SD, *iAUC*_*90*_ mean, *iAUC*_*90*_ SD) were calculated to evaluate intratumoral heterogeneity.

### Immunohistochemical examinations

After completing the final MR study, the implanted tumors were excised. The specimens were examined using H&E and CD31 antibody (1:50 dilution, clone MEC13.3, PharMingen, BD Pharmingen, San Diego, CA) staining of the microvessels. The distribution and morphology of the microvessels in each tumor were assessed under microscopy. Any separable brown immunostained endothelial cell cluster was considered to be a single microvessel. Microvessels in the area of most intense neovascularization were counted in three randomly chosen 200X magnification fields, and the average of the three readings was defined as the microvessel density (MVD). For the apoptosis assay, the samples were stained with cleaved Parp (1:100 dilution, 04–576, Millipore, Billerica, MA). The proportion of positive cells was measured using the Aperio Color Deconvolution Algorithm (Aperio Technologies Inc, Vista, CA).

### Statistical analysis

All statistical analyses were performed using R software version 3.0.2 (R Foundation for Statistical Computing, Vienna, Austria). A two-sided *p* value ≤ 0.05 was considered to indicate statistical significance. The distributional properties of continuous variables were expressed by mean ± standard deviation (SD). The percentage of treatment response was defined as the percentage of change in tumor volume (mm^3^) between baseline and final evaluations. Differences in treatment responses between each group classified by tumor size and microvessel density were assessed using the Wilcoxon rank-sum test depending on the result of Shapiro-Wilk test of normality.

## Results

**Fig 1:**
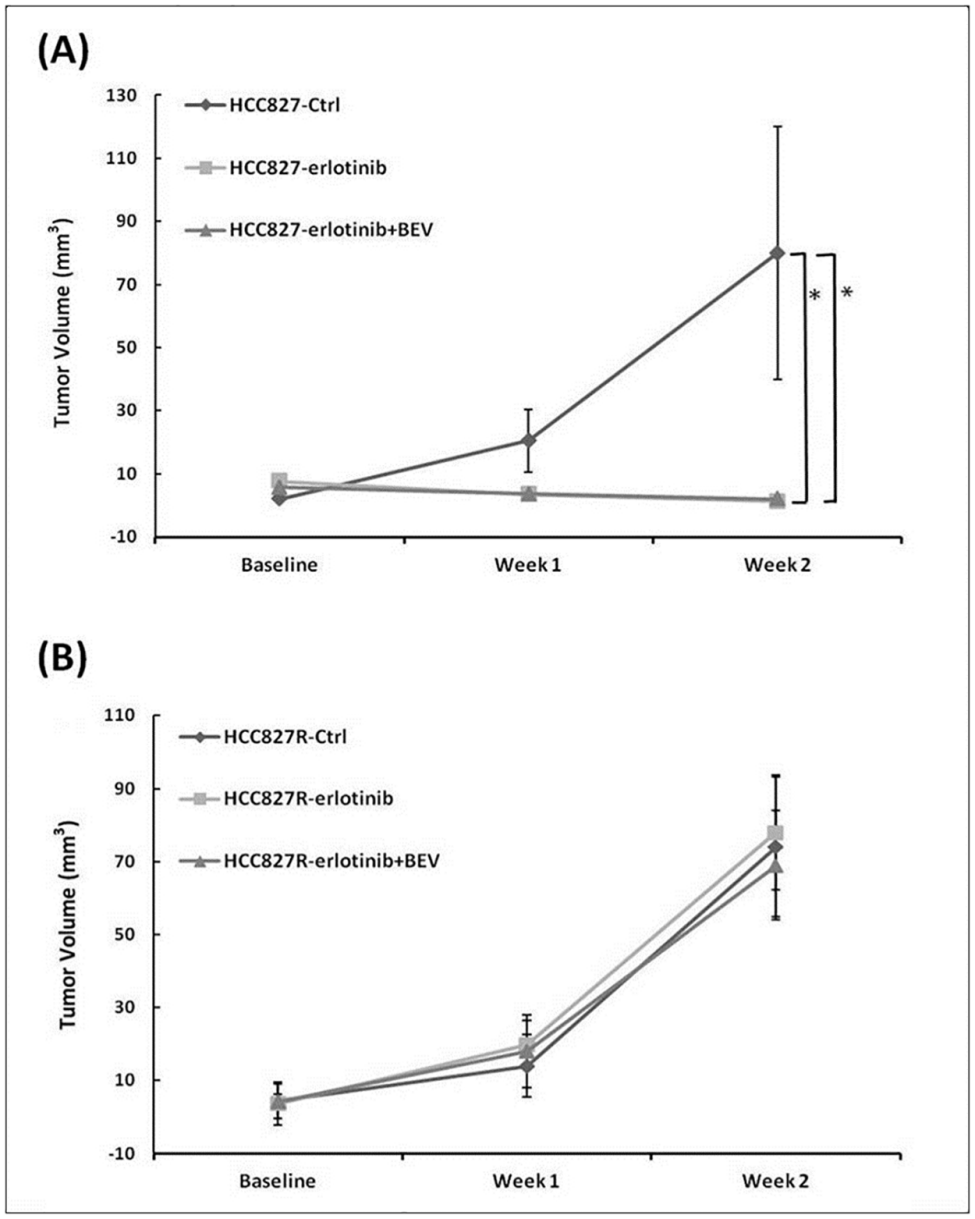
Plots of tumor volume among the control, erlotinib, and combined erlotinib and bevacizumab (BEV) groups in HCC827 (A) and HCC827R (B) mice before and after treatment. Data are presented as the mean ± SD from week 0 (baseline) to week 2. *p < 0.05.

MRI studies of the gefitinib-sensitive (HCC827) and gefitinib-resistant (HCC827R) lung cancer mice were carried out weekly according to three different regimens (erlotinib alone, erlotinib combined with bevacizumab, and controls without treatment). The average values of DCE parameters and ADC for each group are listed in Tables 1 and 2. Continuous tumor growth was observed in the control group, whereas the tumor size decreased in the erlotinib alone and erlotinib combined with bevacizumab groups in the gefitinib-sensitive tumor model (Fig 1A), with reductions in tumor volume of 79% and 56%, respectively, after 2 weeks of treatment (p = 0.008 and 0.006). Rapid tumor growth was noted in both treatment groups in the gefitinib-resistant lung cancer model with no significant differences (Fig 1B).

**Table 1:** Comparison of the DCE and DW MR parameters among control, erlotinib, and combined erlotinib and bevacizumab (BEV) groups in HCC827.

HCC 827	Baseline	Week 1	Week 2
***iAUC***_***90***_			
**Ctrl**	142.4 ± 28.4	188.1 ± 112.0	323.4 ± 88.7
**erlotinib**	353.2 ± 103.3	306.6 ± 118.6	196.2 ± 90.5^†^
**erlotinib+BEV**	391.4 ± 176.0	180.4 ± 73.7*^‡^	122.7 ± 68.6*
***K***^***trans***^ **(min**^**-1**^**)**			
**Ctrl (min**^**-1**^**)**	65.4 ± 19.4	85.2 ± 33.6	159.9 ± 37.8
**erlotinib**	143.7 ± 57.4	115.0 ± 36.1	77.5 ± 49.7^†^
**erlotinib+BEV**	101.7 ± 33.4	85.9 ± 39.8	63.5 ± 43.3*
***k***_***ep***_ **(min**^**-1**^**)**			
**Ctrl**	172.6 ± 33.4	274.9 ± 138.4	716.6 ± 183.2
**erlotinib**	580.8 ± 132.9	356.5 ± 127.2^†^	222.6 ± 132.5^†^
**erlotinib+BEV**	300.3 ± 80.9	241.4 ± 108.2*	163.4 ± 81.6*
**ADC (×10**^**6**^ **mm**^**2**^**)**			
**Ctrl**	1432.4 ± 488.8	1342.8 ± 311.8	1194.7 ± 275.9
**erlotinib**	1170.7 ± 162.7	2071.1 ± 685.9^†^	2992.5 ± 1595.8^†^
**erlotinib+BEV**	1352.9 ± 629.1	1461.9 ± 671.4	1581.4 ± 302.9*
***V***_***e***_			
**Ctrl**	365.3 ± 137.7	534.5 ± 326.7	267.1 ± 89.3
**erlotinib**	477.7 ± 313.6	316.4 ± 93.2	263.9 ± 111.1
**erlotinib+BEV**	362.9 ± 76.0	389.8 ± 173.8	400.6 ± 441.1
***V***_***p***_			
**Ctrl**	98.8 ± 80.6	76.5 ± 44.1	80.1 ± 52.5
**erlotinib**	115.9 ± 67.5	89.5 ± 69.8	68.4 ± 69.2
**erlotinib+BEV**	52.7 ± 25.4	57.3 ± 53.6	98.5 ± 67.8

Ctrl, control (without treatment); BEV, bevacizumab

Data presented were mean absolute value ± standard deviation (S.D.).

The P values were obtained from the paired t-test or Wilcoxon signed-rank test.

^†,^ p <0.05 for comparison between “Ctrl” and “erlotinib”.

*, p < 0.05 for comparison between “Ctrl” and “erlotinib combined with BEV”.

^‡,^ p < 0.05 for comparison between “erlotinib” and “erlotinib combined with BEV”.

**Table 2:** Comparison of the DCE and DW MR parameters among control, erlotinib, and combined erlotinib and bevacizumab (BEV) groups in HCC827R.

HCC 827R	Baseline	Week 1	Week 2
***iAUC***_***90***_			
**Ctrl**	245.1 ± 175.9	358.0 ± 151.6	346.0 ± 99.1
**erlotinib**	212.4 ± 130.4	204.9 ± 73.8	123.6± 67.9
**erlotinib+BEV**	176.8 ± 90.6	174.3 ± 111.8	215.0 ± 134.3
***K***^***trans***^ **(min**^**-1**^**)**			
**Ctrl**	81.2 ± 34.7	123.9 ± 75.9	173.2 ± 101.9
**erlotinib**	86.4 ± 18.2	98.8 ± 73.3	151.8 ± 48.0
**erlotinib+BEV**	59.8 ± 20.4	86.8 ± 59.2	96.3 ± 50.7
***k***_***ep***_ **(min**^**-1**^**)**			
**Ctrl**	226.3 ± 154.3	612.7 ± 636.1	563.8 ± 363.5
**erlotinib**	281.2 ± 128.6	359.6 ± 246.3	800.8 ± 737.8
**erlotinib+BEV**	137.6 ± 66.4	501.4 ± 414.8	455.5 ± 205.2
**ADC(×10**^**6**^**mm**^**2**^**)**			
**Ctrl**	1393.5 ± 414.1	628.1 ± 326.7	387.5 ± 214.9
**erlotinib**	1537.6 ± 417.5	325.6 ± 133.2	623.0 ± 741.7
**erlotinib+BEV**	1565.6 ± 420.6	315.2 ± 229.8	239.2 ± 100.3
***V***_***e***_			
**Ctrl**	472.3 ± 371.3	628.1 ± 326.7	387.5 ± 214.9
**erlotinib**	411.7 ± 166.5	325.6 ± 133.2	623.0 ± 741.7
**erlotinib+BEV**	487.8 ± 185.4	315.2 ± 229.8	239.2 ± 100.3
***V***_***p***_			
**Ctrl**	116.5 ± 84.5	115.2 ± 104.4	92.3 ± 31.3
**erlotinib**	77.0 ± 30.9	71.2 ± 50.8	67.7 ± 51.8
**erlotinib+BEV**	94.0 ± 62.8	53.9 ± 36.6	69.1 ± 47.0

**Fig 2:**
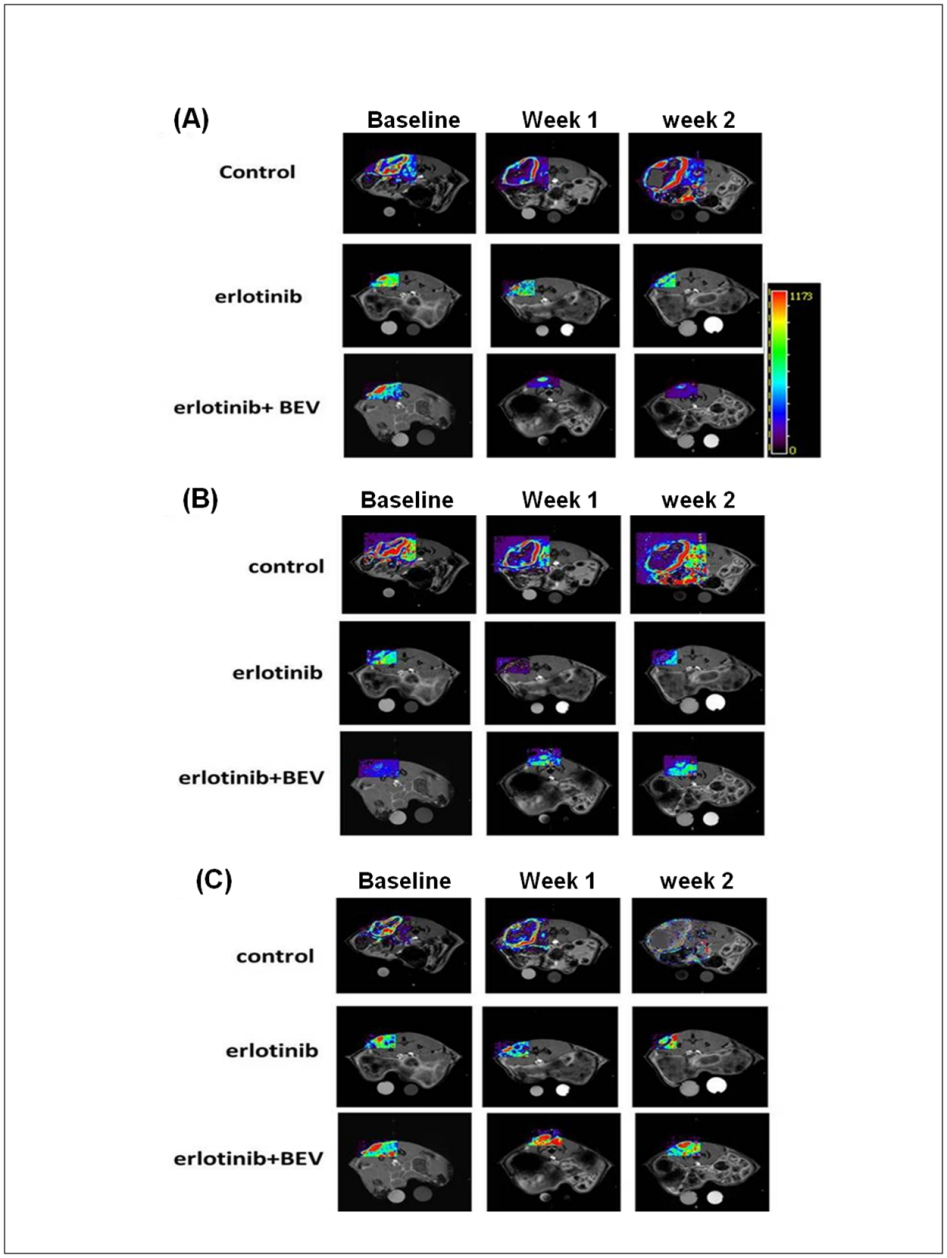
MR-DCE images and parametric maps from representative mice in the HCC827 model receiving different treatment regimens. (A) *K*^*trans*^, (B) *k*_*ep*_ and (C) *iAUC*_*90*_ mapping of one representative mouse from each group before and after treatment.

Interestingly, decreases in the DCE-MR derived parameters (such as *K*^*trans*^, *k*_*ep*_ and *iAUC*_*90*_) at different time points in the HCC827 mice model (gefitinib sensitive) were found along with the decreases in tumor size (Fig 2). However, in the gefitinib-sensitive model, no significant changes in the parameters were noted in the gefitinib-resistant model (data not shown).

**Fig 3:**
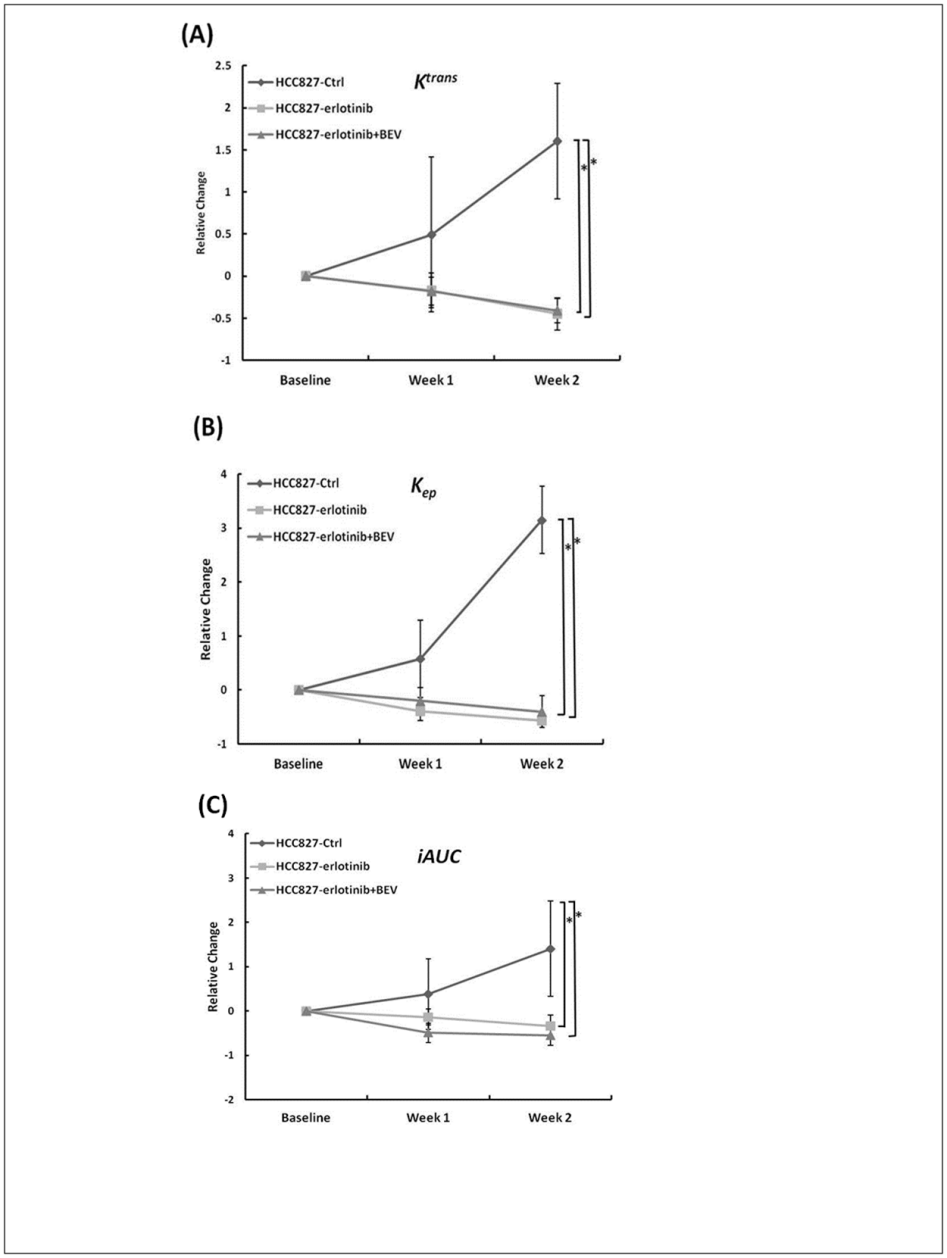
Plots of relative changes in DCE parameters for each group in HCC827 mice. The parameters, (A) *K*^*trans*^, (B) *k*_*ep*_ and (C) *iAUC*_*90*_ are shown from week 0 (baseline) to week 2.

Group plots of relative changes in each group from baseline to week 2 are shown in Fig 3. *K*^*trans*^ significantly decreased at week 2 in the erlotinib alone and combined with bevacizumab groups (p = 0.007 and 0.004) and significant decreases in *k*_*ep*_ in the erlotinib alone and combined with bevacizumab groups were detected even earlier at week 1 (p = 0.008 and 0.03) and week 2 (p = 0.008 and 0.004). Similarly, significant decreases in *iAUC*_*90*_ were observed at week 2 in the erlotinib alone (p = 0.008) and combined with bevacizumab groups (p = 0.004). In addition, there was a significant difference in *iAUC*_*90*_ between the erlotinib alone and combined with bevacizumab group at week 1 (p = 0.03), even though no significant difference in tumor volume was found at this time point in either treatment group. However, no significant changes in DCE parameters were noted in the gefitinib-resistant model (data not shown).

**Fig 4:**
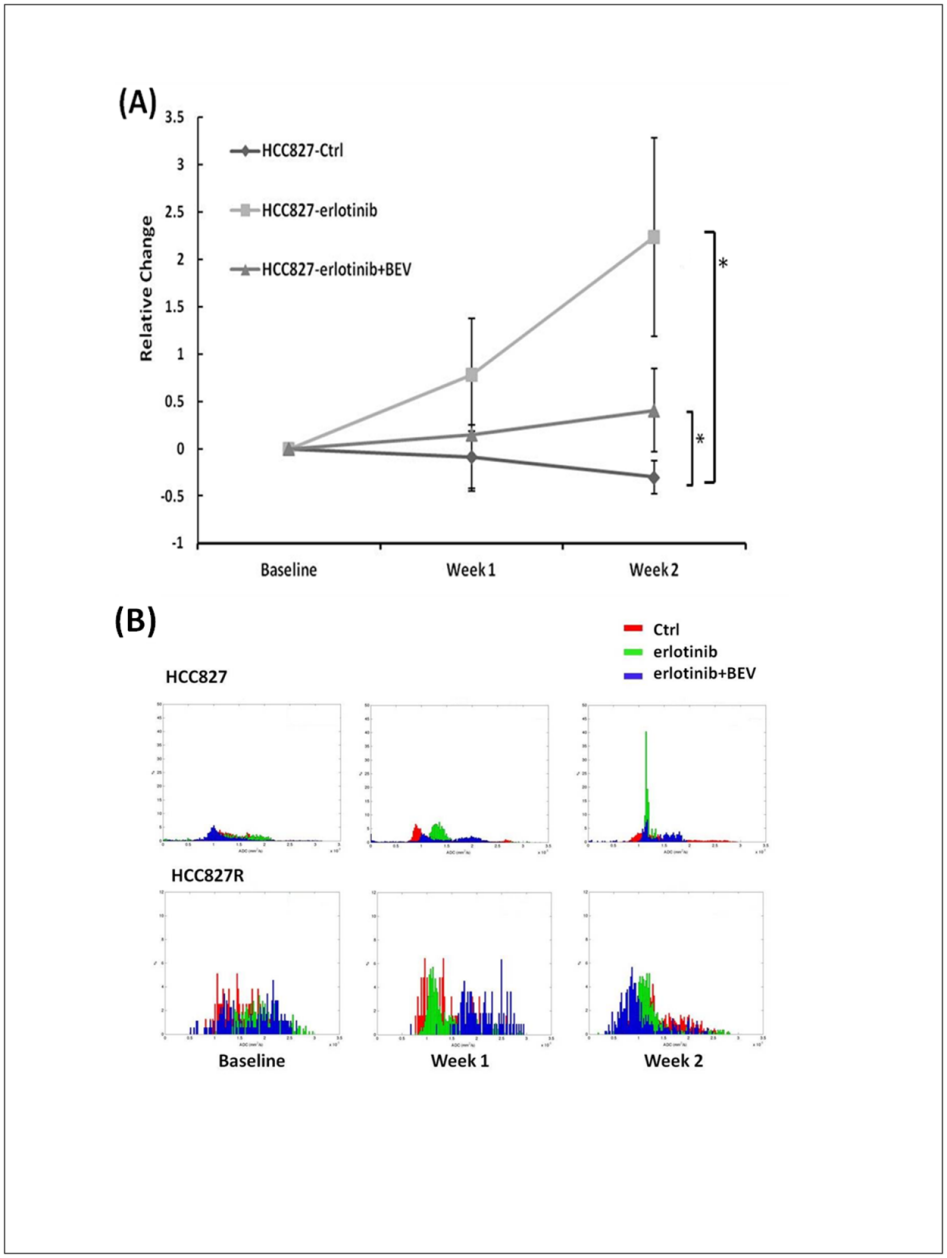
Changes of MR apparent diffusion coefficient parameter: ADC. (A) Relative changes and (B) normalized histograms of ADC for each group in the HCC827 and HCC827R lung cancer mice.

The response to treatment was evaluated using DW-MRI, and the results of the percentage change in ADC in group analysis are shown in Fig 4A. The mice in the gefitinib-sensitive lung cancer model treated with erlotinib had significant increases in ADC at week 1 and week 2 (p = 0.04 and 0.03) and those in the erlotinib combined with bevacizumab group had a significant increase at week 2 (p = 0.045), whereas the control group showed a decreased in ADC as the tumors grew. Normalized histograms of the three groups also indicated a different peak shift after treatment (Fig 4B), with peak shifts toward higher ADC values at week 1 and then transiently at week 2 in both treatment groups in the gefitinib-sensitive lung cancer model. However, in the gefitinib-resistant model, ADC decreased in all treatment groups during the follow-up period, and there were no consistent peak changes as the tumors grew. In addition, there were no significant changes in ADC values in DW-MRI between the treatment groups as with DCE-MRI in the gefitinib-resistant NSCLC model.

**Fig 5:**
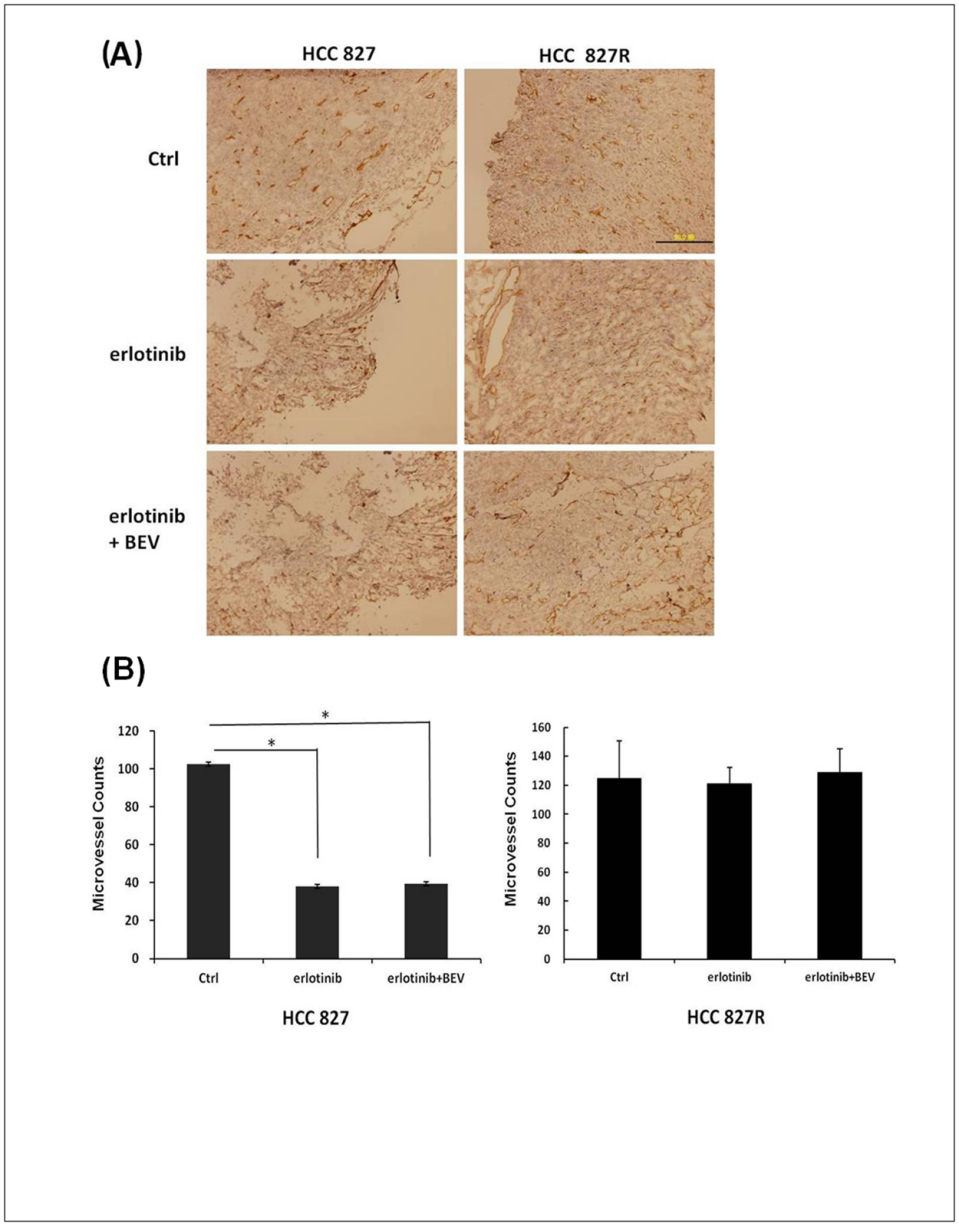
Histological staining of tumor vessels in the HCC827 and HCC827R lung cancer mice tissues. (A) Representative slides stained with anti-CD31 antibodies for each group (200X). (B) MVD was counted in three randomly chosen 200X. Columns, mean; Bars, SD. *p < 0.05.

Normalized histograms of ADC showed higher ADC values in the tumors of the treatment groups compared to the control group, which corresponded to the histologic findings in the TKI-sensitive tumor model. Percentage reductions in the DCE parameters (*K*^*trans*^, *k*_*ep*_, and *iAUC*_*90*_; Figs 1–3) were correlated with the histologic findings of MVD (Fig 5). Significant differences in MVD were detected using the endothelial cell marker CD31 in the erlotinib group versus the control group (p = 0.007) and erlotinib combined with bevacizumab group versus the control group (p = 0.004) in the gefitinib-sensitive tumor model (Fig 5A). However, there were no significant differences in MVD between the different regimens in the gefitinib-resistant model (Fig 5B).

**Fig 6:**
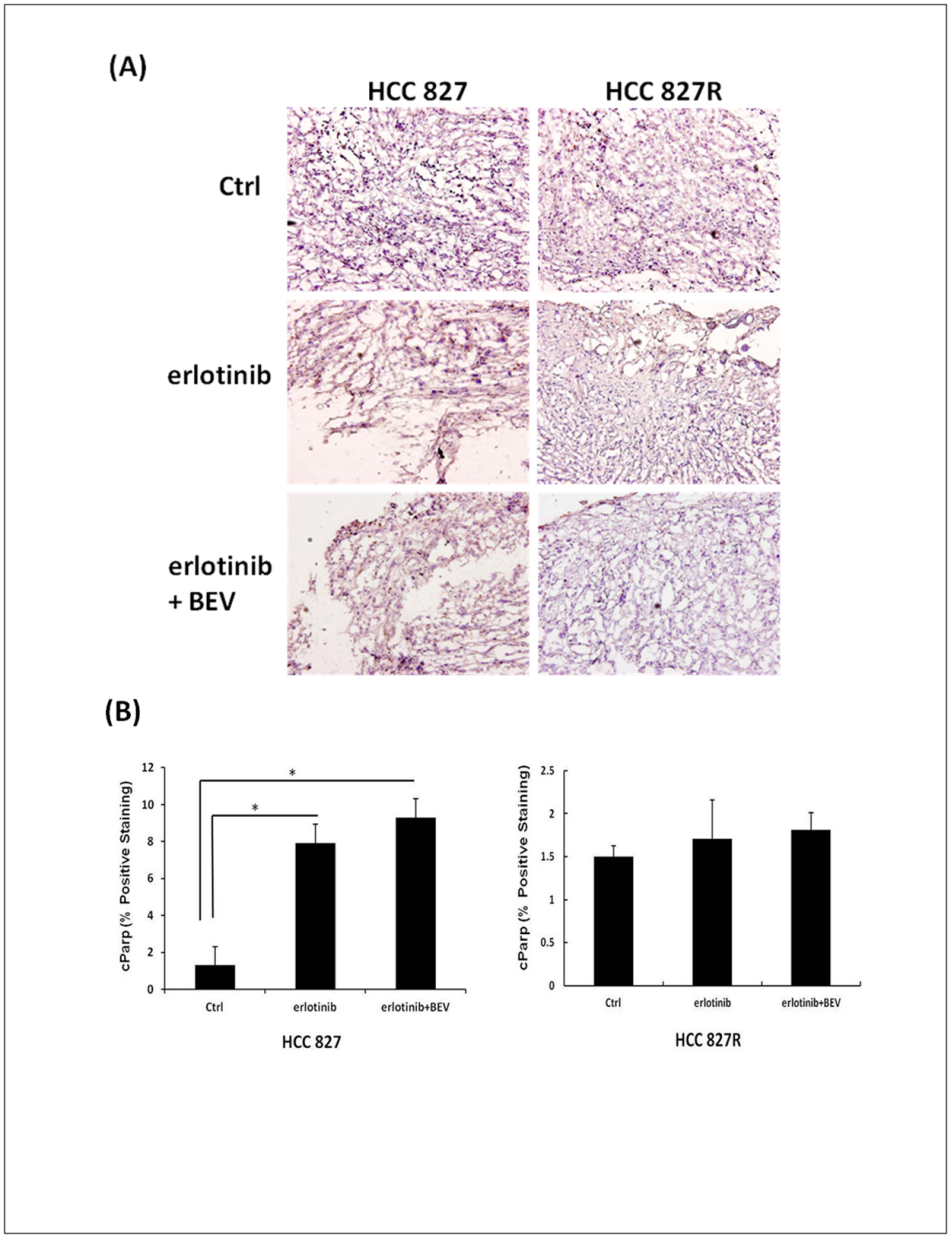
Samples stained for cParp for each group in the HCC827 and HCC827R lung cancer mice. (A) The brown staining of cParp images in the treatment groups of the HCC827 mice indicated the positive cells undergoing apoptosis. (B) Percentage of cParp-positive pixels. cParp, cleaved Poly (ADP-ribose) polymerase. Columns, mean; Bars, SD. *p < 0.05.

A variable degree of tumor necrosis was observed in the histologic staining of the mice in the HCC827 model that received erlotinib or erlotinib combined with bevacizumab (Fig 5A). Areas of central tumor necrosis corresponded to higher ADC values within the tumors. The control group showed prominent tumor vascularity and lower necrotic fractions (Fig 5A). In addition, we also found significantly increased tumor apoptosis after analyzing the cellular activity of cleaved Parp, a marker of apoptosis (Fig 6). A statistically significant increase in apoptosis was found with the effect of a therapeutic response in the erlotinib group (p = 0.007) and erlotinib combined with bevacizumab group (p = 0.004) compared with the control group in the gefitinib-sensitive lung cancer model.

## Discussion

EGFR-TKIs have been shown to have significant clinical therapeutic effects in patients with lung cancer who harbor EGFR activating mutations [38]. Recently, target therapy alone or combined with chemotherapy has been used to treat advanced NSCLC, and drugs targeting the EGFR and VEGF pathways have become new therapeutic options for lung cancer therapy. In the HCC827 xenograft model of the present study, erlotinib alone and erlotinib combined with bevacizumab considerably suppressed tumor growth compared with control groups, and erlotinib in particular elicited an extremely strong tumor inhibition at the dose of 25 mg/kg/day. The inhibition of erlotinib combined with bevacizumab was as efficient as that of erlotinib alone may be associated with the high dose of erlotinib. Similar results were reported by Furugaki et al., however, by reducing erlotinib to 5 mg/kg/day, they showed an additive inhibitory effect of erlotinib and bevacizumab combination compared to erlotinib alone in a 22-day treatment period in HCC827 xenografts [40]. Therefore, we performed another EGFR TKI-sensitive PC9 xenograft model and examined the effects of low dose EGFR-TKI (gefitinib, 10 mg/kg/day) and gefitinib plus bevacizumab. The further results indicated that bevacizumab combination significantly enhanced antitumor activity of gefitinib as shown in the supporting information (S1 and S4 Figs). DCE and DW MRI derived parameters were also associated with the therapeutic response in the TKI-sensitive model (S2 and S3 Figs).

The current clinical standard for assessing treatment response is to measure changes in lesion size [33]. However, the use of changes in size is limited when assessing tumor response, particularly when assessing newer cancer therapies that can stabilize the disease [21–23]. It is known that a change in the size of a tumor may be delayed chronologically, and thus several courses of treatment are often required before a conclusion can be made regarding the response of a tumor to treatment [23]. DCE-MRI provides important pharmacokinetic parameters which can measure sensitive pathophysiological characteristics and detect changes in tumor vasculature and the peritumoral microenvironment [27–28]. In the current study, the same inhibitory effects of erlotinib alone and erlotinib combined with bevacizumab groups in the gefitinib-sensitive tumor model were observed at week 1 as the tumor size decreased shown in Fig 1A but no significant differences were detected between the two treatment groups. However, the value of *iAUC*_*90*_ was reduced more in combination groups than erlotinib alone at this time point (Table 1). In addition, the relative change of *iAUC*_*90*_ (Fig 3C) between the two treatment groups at week 1 was significant (p = 0.03). The changes of *iAUC*_*90*_ were really earlier than that of tumor volume between the two treatment groups in the gefitinib-sensitive model. Imaging the tumor physiology allowed us to detect changes in *iAUC*_*90*_ before volumetric changes between the erlotinib and erlotinib combined with bevacizumab groups. Therefore, the therapeutic response to the two target treatments could be assessed by MRI in the early phase of tumor development.

DW-MRI evaluates the diffusion of water molecules, and it has been shown to be correlated with cell density measurements in cancer models [31, 32, 41]. Using non-invasive MRI imaging with DCE and ADC quantification, early pathological changes could be observed, and the color mapping provided further information on the internal components and vascular structures of tumors, and this may allow for sensitive and rapid evaluations during tumor progression.

In this study, the pharmacokinetic analysis of DCE-MRI was significantly correlated with effective tumor control and vascular inhibition, consistent with previous reports in which most tumors had a decrease in *K*^trans^ after weeks of treatment, indicating an inhibitory effect on tumor vasculature [42–43]. These findings support the hypothesis that the more sensitive imaging tools DCE and DW-MRI should be used when evaluating tumor response. The imaging signals and distribution of the imaging components allowed for the analysis of the therapeutic effects from DCE and DW-MRI, and the findings corresponded to the histologic findings in this study. Among the parameters derived from DCE-MRI in this study, *K*^*trans*^, *k*_*ep*_ and *iAUC*_*90*_ decreased from week 1 in the treatment groups compared to the control group. This suggests that a decrease in *k*_*ep*_ is the result of an expanded extracellular extravascular space of the treated tumors, and that a decrease in *iAUC*_*90*_ indicates the reduction of total tumor vascularity and vascular function.

However, no effective tumor control was observed using the TKI even when combined with anti-angiogenesis therapy in the gefitinib-resistant NSCLC xenografts. Mechanisms of acquired resistance to EGFR-TKI in NSCLC are secondary mutations in EGFR itself, including the EGFR T790M mutation (about 50%) and amplification of the MET oncogene (15–20%) [44–45]. Engelman et al. had found overexpression of MET in HCC827 cells was sufficient to confer resistance and suggested that MET amplified gefitinib resistant cells (HCC827R) utilize ERBB3 as the primary adaptor to activate PI3K/Akt signaling in the presence of EGFR TKIs [39]. The identified resistant mechanisms to EGFR-TKIs may provide specific targets for development of effective therapeutic strategies.

In DW-MRI, the higher ADC values of the treated mice in the gefitinib-sensitive model reflected the increased water diffusion that may correspond to loss of tissue and reduction of vessel density [46]. When tumor cells begin to die, increased water mobility cause a peak shift of ADC as seen in the regular target therapy of this study. Several previous studies have also reported that EGFR-TKIs, including gefitinib and erlotinib, induce apoptosis in NSCLC cell lines through the activation of intrinsic pathways [47–48]. The higher ADC values and significant increases in cleaved Parp as shown in the staining results indicate caspase-dependent apoptosis. This provides a more detailed understanding of the mechanisms involved in the pathways of the EGFR-TKI erlotinib and bevacizumab, and this may provide a more rational basis for their therapeutic use.

In this study, we demonstrated an early increase in absolute ADC values and decreases in *K*^*trans*^, *k*_*ep*_ and *iAUC*_*90*_ in treatment with both TKI alone and in combination with an anti-angiogenesis agent compared to the untreated tumors before apparent changes in tumor size. This final result was shown by decreased vascularity of the treated tumors as reflected by decreased MVD. There are two main types of vascular targeting agents, monoclonal antibodies against VEGF such as bevacizumab as used in the study, and other TKIs such as sunitinib, vandetanib, and sorafenib [49]. Different kinds of VEGF TKIs have certain unique characteristics including activity against TK domains of several other receptors involved in tumor proliferation such as c-Kit, Raf, PDGF, and FGF, and this can lead to the selective inhibition of new vessel growth and decreased vascular perfusion and permeability in tumors. In this study, the lack of antiangiogenesis efficacy in the combination therapy probably reflects the redundancy of proangiogenic pathways other than the vascular epidermal growth factor pathway [50] and the highly heterogeneous nature of NSCLC. In addition, it is necessary to identify predictive factors of efficacy for bevacizumab and erlotinib. This is probably the real challenge to optimize the use of these drugs. Further studies are needed to investigate whether these findings can be extended to other antiangiogenesis agents and to elucidate different properties of MRI parameters, and potentially the development of new management strategies.

Our results suggest the usefulness of early increases in ADC and quantitative changes in the DCE parameters *K*^*trans*^, *k*_*ep*_ and *iAUC*_*90*_ as pharmacodynamic biomarkers for tumor response to effective therapy and improvements with the additional therapeutic options. DCE-MRI has been widely used to assess vascular perfusion and permeability by pharmacokinetic modeling of tissues in patients with different cancers [28]. Therefore, further technical advances in arterial input function identification, kinetic model improvement, and new image acquisition technology are needed before DCE-MRI can be used routinely in clinical oncology. However, there are several limitations when evaluating DCE-MRI parameters. *iAUC*_*90*_ relies on an accurate T1 map and represents the composition of physiologic processing, but it may not accurately reflect vascular changes. In addition, *K*^*trans*^ is dependent on determining arterial input function and may be more physiologically meaningful.

This study confirmed that changes in tumor size after treatment were related to its characteristics, and that selecting the most appropriate target drug can be effective in inhibiting tumor growth. The intrinsic composition of the tumor, including vascular perfusion and permeability characteristics were associated with changes in tumor size, and they could be obtained by MRI and proven by pathology and immunostaining. These findings provided basic evidence for clinical studies of lancet oncology in 2014 [18]. Two weeks of treatment may be too short a time to determine the effects of bevacizumab in this study. Usually, the effects of bevacizumab take several weeks (more than 21 days) to consolidate, and thus, the initial therapeutic response of bevacizumab which have lasted at most two weeks, may be too short to assess efficacy of additive antitumor activity. In this case, it is reasonable to prolong the treatment period to 3–4 weeks to further evaluate the therapeutic effects of combination and assess the long-term survival of the animals. Combining the findings of MRI and immunohistochemical quantification, our results confirmed the suppression of tumor growth after treatment with the two targeted agents. In this study, there was a limitation in observing a longer period of treatment effect because of the relatively large tumor growth compared to the small body size of the mice.

## Supporting information

S1 Fig Tumor growth of PC9 xenografts with low-dose EGFR TKI + BEV therapy. Mice bearing PC9 xenografts were treated 10 mg/kg of gefitinib or gefitinib (10 mg/kg) +BEV (5 mg/kg), n = 4–5. Data are presented as the mean ± SD from week 0 (baseline) to week 2. *p < 0.05.
(PDF)

S2 Fig Plots of relative changes and color maps of DCE parameters for each group in PC9 xenografts. The parameters: (A) and (C) *K*^*trans*^, (B) and (D) *iAUC*_*90*_ are shown from week 0 (baseline) to week 2. Data are presented as the mean ± SD, *p < 0.05.
(PDF)

S3 Fig Changes of MR apparent diffusion coefficient parameter: ADC. (A) Relative changes and (B) normalized histograms of ADC for each group in PC9 xenografts.
(PDF)

S4 Fig Histological staining of tumor vessels in PC9 mice tissues. (A) Representative immunohistochemical images of CD31-immunostaining for each group (100X). (B) MVD in tumor tissue was counted in three randomly chosen. Columns, mean; Bars, SD.
(PDF)
