# Supplementary material for: Functional evaluation of therapeutic response of HCC827 lung cancer to bevacizumab and erlotinib targeted therapy using dynamic contrast-enhanced and diffusion-weighted MRI
Source: PLoS One. 2017 Nov 9;12(11):e0187824. doi: 10.1371/journal.pone.0187824 (PMC5679602; doi:10.1371/journal.pone.0187824)
Supplement: S1 Fig — Mice bearing PC9 xenografts were treated 10 mg/kg of gefitinib or gefitinib (10 mg/kg) +BEV (5 mg/kg), n = 4–5. Data are presented as the mean ± SD from week 0 (baseline) to week 2. *p < 0.05. (PDF) [file pone.0187824.s001.pdf]

Tumor growth of PC9 xenografts with low-dose EGFR TKI + BEV therapy. Mice bearing PC9 xenografts were treated 10 mg/kg of gefitinib or gefitinib (10 mg/kg) +BEV (5 mg/kg), n=4-5. Data are presented as the mean $\pm$ SD from week 0 (baseline) to week 2. *$p < 0.05$

S1 Fig

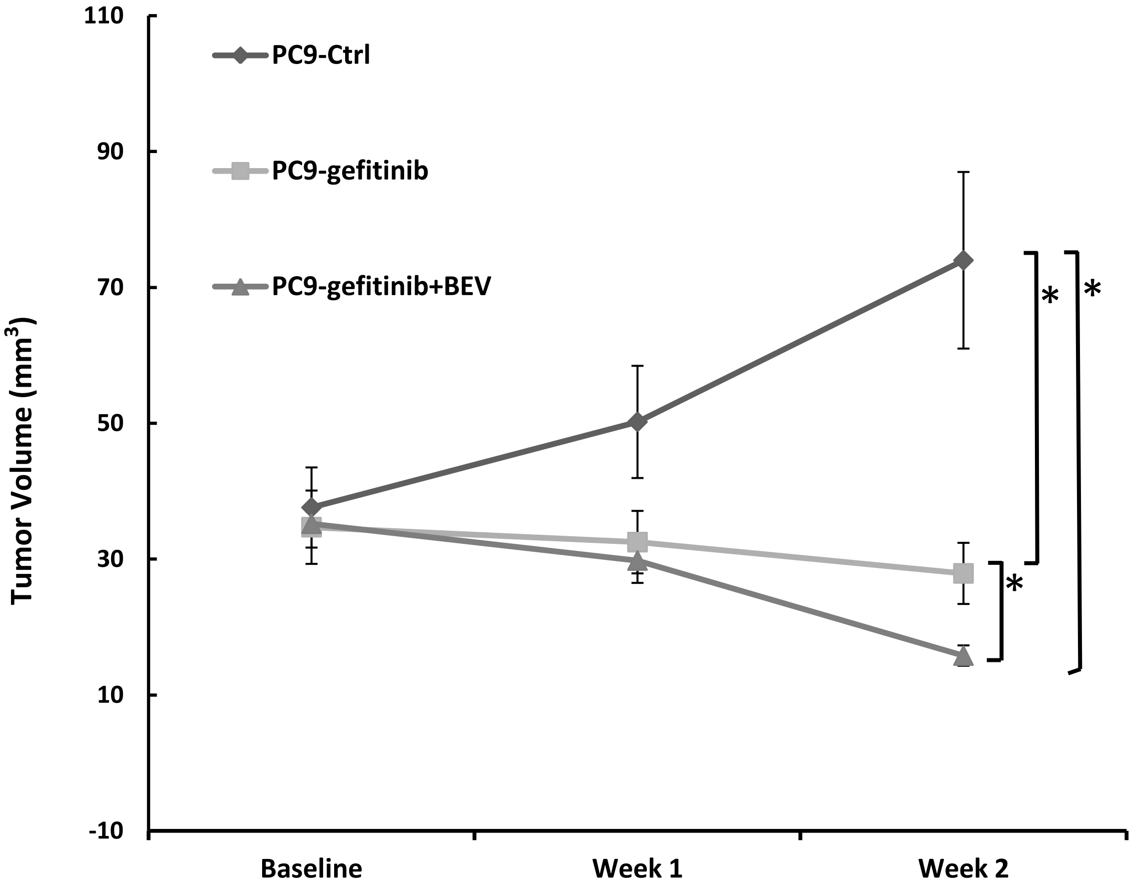
PC9-Ctrl
PC9-gefitinib
PC9-gefitinib+BEV
Tumor Volume (mm3)
110
90
70
50
30
10
-10
Baseline
Week 1
Week 2
*
*
*
