# Supplementary material for: Functional evaluation of therapeutic response of HCC827 lung cancer to bevacizumab and erlotinib targeted therapy using dynamic contrast-enhanced and diffusion-weighted MRI
Source: PLoS One. 2017 Nov 9;12(11):e0187824. doi: 10.1371/journal.pone.0187824 (PMC5679602; doi:10.1371/journal.pone.0187824)
Supplement: S2 Fig — The parameters: (A) and (C) Ktrans, (B) and (D) iAUC90 are shown from week 0 (baseline) to week 2. Data are presented as the mean ± SD, *p < 0.05. (PDF) [file pone.0187824.s002.pdf]

Plots of relative changes and color maps of DCE parameters for each group in PC9 xenografts. The parameters: (A) and (C) K^{trans}, (B) and (D) $iAUC_{90}$ are shown from week 0 (baseline) to week 2. Data are presented as the mean $\pm$ SD ,*$p < 0.05$

S2 Fig

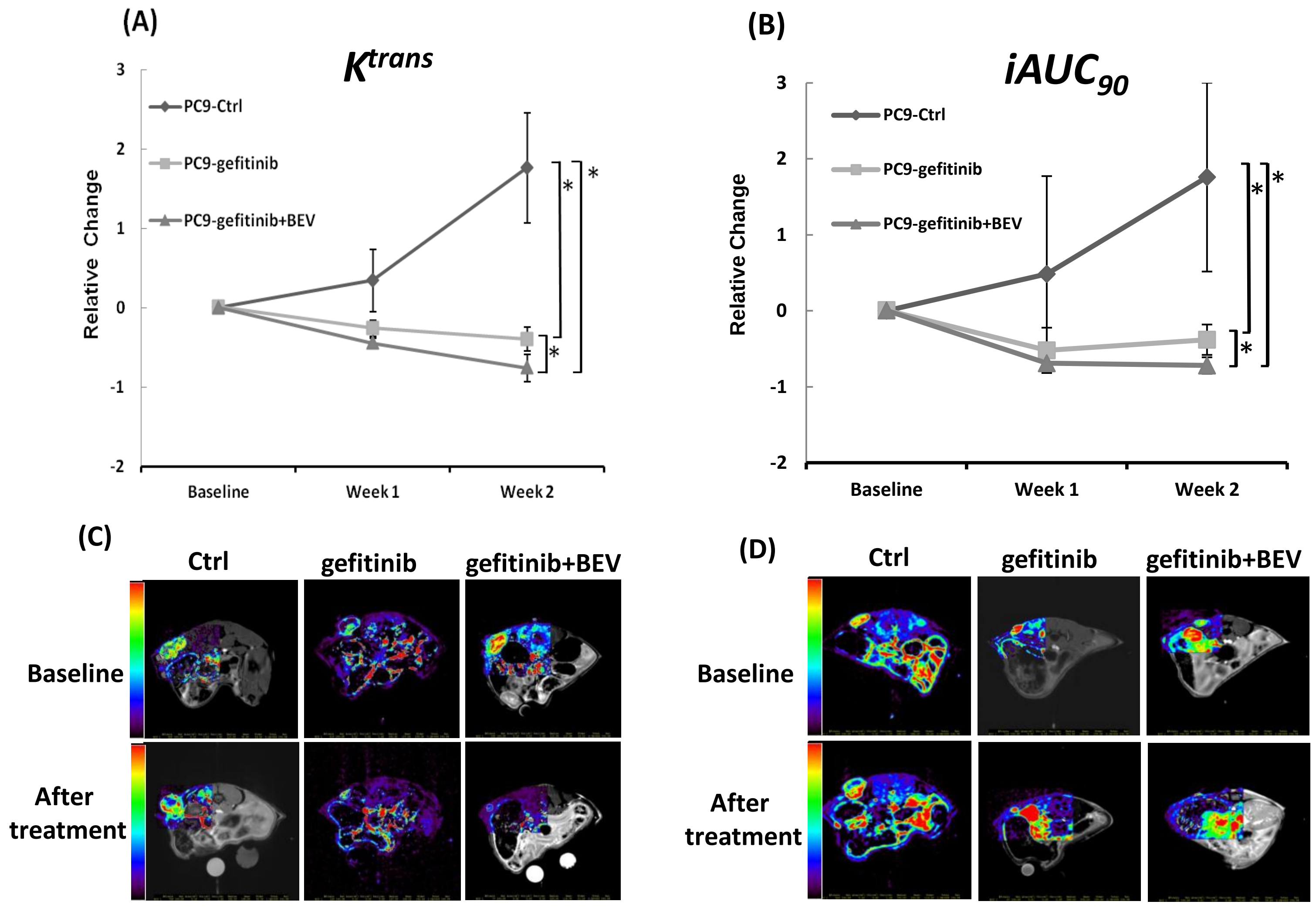

(A)
K^{trans}
PC9-Ctrl
PC9-gefitinib
PC9-gefitinib+BEV
Relative Change
3
2
1
0
-1
-2
Baseline
Week 1
Week 2
*
*
*
(B)
$iAUC_{90}$
PC9-Ctrl
PC9-gefitinib
PC9-gefitinib+BEV
Relative Change
Baseline
Week 1
Week 2
(C)
Ctrl
gefitinib
gefitinib+BEV
Baseline
After treatment
(D)
Ctrl
gefitinib
gefitinib+BEV
Baseline
After treatment
