# Supplementary material for: Functional evaluation of therapeutic response of HCC827 lung cancer to bevacizumab and erlotinib targeted therapy using dynamic contrast-enhanced and diffusion-weighted MRI
Source: PLoS One. 2017 Nov 9;12(11):e0187824. doi: 10.1371/journal.pone.0187824 (PMC5679602; doi:10.1371/journal.pone.0187824)
Supplement: S3 Fig — (A) Relative changes and (B) normalized histograms of ADC for each group in PC9 xenografts. (PDF) [file pone.0187824.s003.pdf]

Changes of MR apparent diffusion coefficient parameter: ADC.
(A) Relative changes and (B) normalized histograms of ADC for each group in PC9 xenografts.

S3 Fig

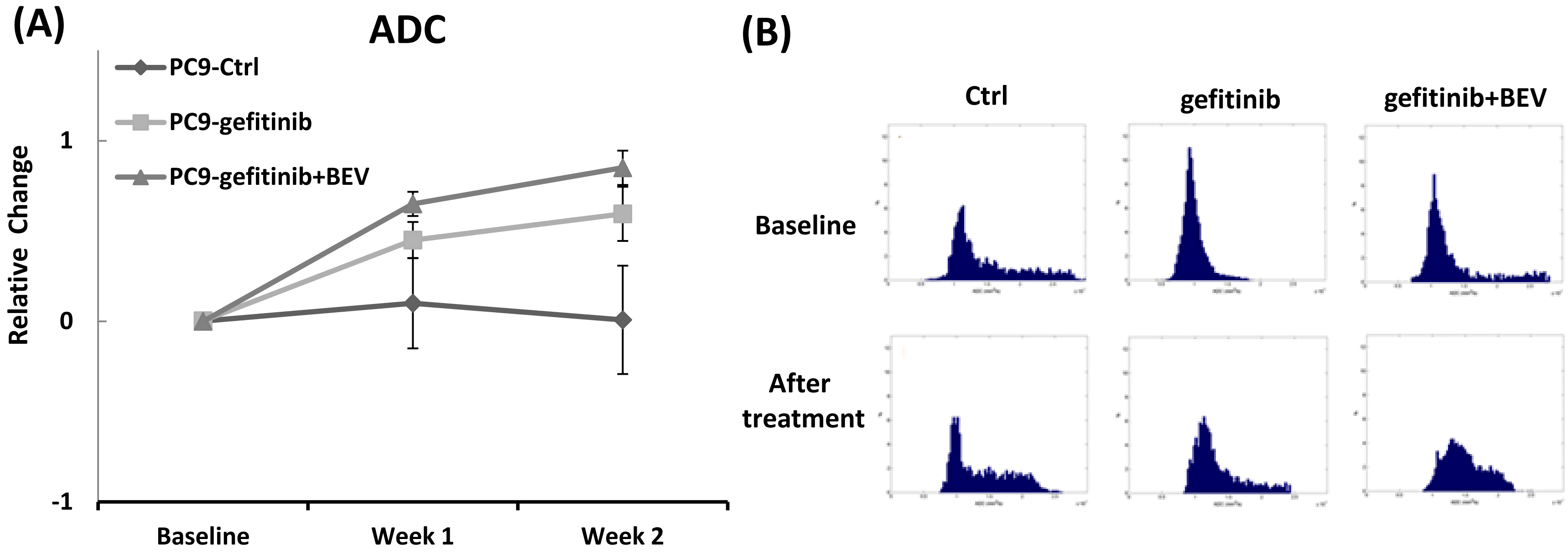

(A)
ADC
PC9-Ctrl
PC9-gefitinib
PC9-gefitinib+BEV
Relative Change
1
0
-1
Baseline
Week 1
Week 2
(B)
Ctrl
gefitinib
gefitinib+BEV
Baseline
After treatment
