# Supplementary material for: Functional evaluation of therapeutic response of HCC827 lung cancer to bevacizumab and erlotinib targeted therapy using dynamic contrast-enhanced and diffusion-weighted MRI
Source: PLoS One. 2017 Nov 9;12(11):e0187824. doi: 10.1371/journal.pone.0187824 (PMC5679602; doi:10.1371/journal.pone.0187824)
Supplement: S4 Fig — (A) Representative immunohistochemical images of CD31-immunostaining for each group (100X). (B) MVD in tumor tissue was counted in three randomly chosen. Columns, mean; Bars, SD. (PDF) [file pone.0187824.s004.pdf]

Histological staining of tumor vessels in PC9 mice tissues.
(A) Representative immunohistochemical images of CD31-immunostaining for each group (100X). (B) MVD in tumor tissue was counted in three randomly chosen. Columns, mean; Bars, SD.

S4 Fig

(A)

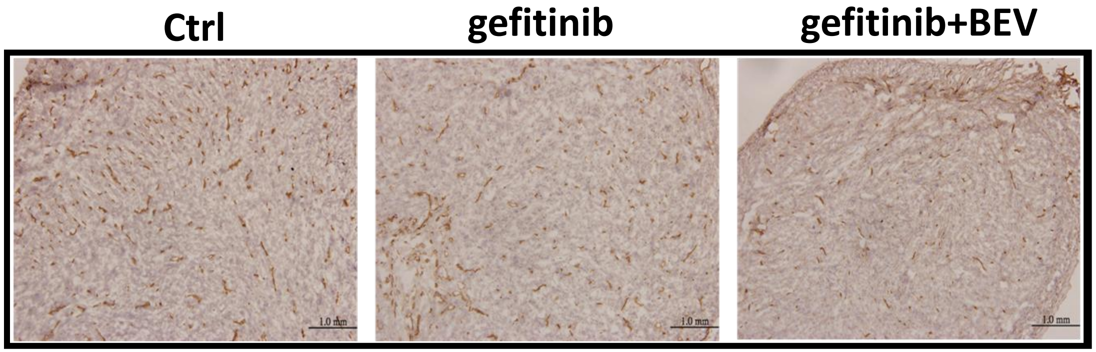
Ctrl
gefitinib
gefitinib+BEV
1.0 mm
1.0 mm
1.0 mm

(B)

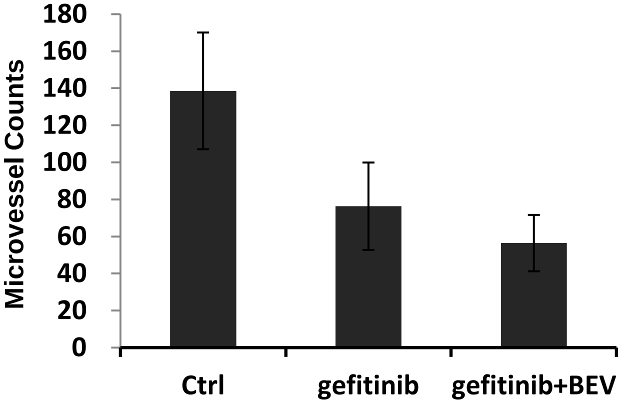
Microvessel Counts
180
160
140
120
100
80
60
40
20
0
Ctrl
gefitinib
gefitinib+BEV
